# Lack of chemopreventive effects of P2X7R inhibitors against pancreatic cancer

**DOI:** 10.18632/oncotarget.22085

**Published:** 2017-10-26

**Authors:** Altaf Mohammed, Naveena B. Janakiram, Venkateshwar Madka, Gopal Pathuri, Qian Li, Rebekah Ritchie, Laura Biddick, Hannah Kutche, Yuting Zhang, Anil Singh, Hariprasad Gali, Stan Lightfoot, Vernon E. Steele, Chen S. Suen, Chinthalapally V. Rao

**Affiliations:** ^1^ Center for Cancer Prevention and Drug Development, Department of Medicine, Hematology-Oncology Section, Stephenson Cancer Center, University of Oklahoma Health Sciences Center, Oklahoma City, OK, USA; ^2^ College of Pharmacy, University of Oklahoma Health Sciences Center, Oklahoma City, OK, USA; ^3^ Chemopreventive Agent Development Research Group, Division of Cancer Prevention, National Cancer Institute, Bethesda, MD, USA; ^4^ VA Medical Center, Oklahoma City, OK, USA; ^5^ Current address: Chemopreventive Agent Development Research Group, Division of Cancer Prevention, National Cancer Institute, Bethesda, MD, USA

**Keywords:** pancreatic cancer, chemoprevention, P2X7R, A438079, AZ10606120

## Abstract

Pancreatic cancer (PC) is an almost uniformly lethal disease with inflammation playing an important role in its progression. Sustained stimulation of purinergic receptor P2X7 drives induction of NLRP inflammasome activation. To understand the role of P2X7 receptor and inflammasome, we performed transcriptomic analysis of p48^Cre/+^-LSL-Kras^G12D/+^ mice pancreatic tumors by next generation sequencing. Results showed that P2X7R's key inflammasome components, IL-1β and caspase-1 are highly expressed (*p* < 0.05) in pancreatic tumors. Hence, to target P2X7R, we tested effects of two P2X7R antagonists, A438079 and AZ10606120, on pancreatic intraepithelial neoplasms (PanINs) and their progression to PC in p48^Cre/+^-LSL-Kras^G12D/+^ mice. Following dose optimization studies, for chemoprevention efficacy, six-week-old p48^Cre/+^-LSL-Kras^G12D/+^ mice (24–36/group) were fed modified AIN-76A diets containing 0, 50 or 100 ppm A438079 and AZ10606120 for 38 weeks. Pancreata were collected, weighed, and evaluated for PanINs and PDAC. Control diet-fed male mice showed 50% PDAC incidence. Dietary A438079 and AZ10606120 showed 60% PDAC incidence. A marginal increase of PanIN 3 (carcinoma *in-situ*) was observed in drug-treated mice. Importantly, the carcinoma spread in untreated mice was 24% compared to 43–53% in treatment groups. Reduced survival rates were observed in mice exposed to P2X7R inhibitors. Both drugs showed a decrease in caspase-3, caspase-1, p21 and Cdc25c. Dietary A438079 showed modest inhibition of P2X7R, NLRP3, and IL-33, whereas AZ10606120 had no effects. In summary, targeting the P2X7R pathway by A438079 and AZ10606120 failed to show chemopreventive effects against PC and slightly enhanced PanIN progression to PDAC. Hence, caution is needed while treating high-risk individuals with P2X7R inhibitors.

## INTRODUCTION

Pancreatic cancer (PC) still remains a devastating disease that is almost uniformly lethal. Over 53,000 individuals will be diagnosed, with >43,000 deaths expected in the US alone in 2017 [1]. Since 2004, the rates of pancreatic cancer have increased by 1.5% per year [1]. Pancreatic cancer accounts for about 3% of all cancers in the US and about 7% of all cancer deaths [1]. Pancreatic Intraepithelial Neoplasia (PanIN) are the most common precursors to invasive pancreatic ductal adenocarcinoma (PDAC), rendering them promising targets for intervention, especially in the high-risk population as it takes several years for PanIN progression into ductal adenocarcinoma and acquire metastatic capacity [2–5]. Chronic inflammation is a hallmark of many diseases, including PC and pancreatitis [6]. Purinergic receptor P2X, ligand-gated ion channel 7 (P2X7R) is one of the potent of the membrane receptors responsible for inflammasome activation and release of inflammatory cytokines. The etiology of PC and laboratory investigations have demonstrated that inflammation represents an important role in its carcinogenesis [6–11].

Inflammation can be tumorigenic through multiple molecular mechanisms, including elevated inducible nitric-oxide synthase (iNOS), cyclooxygenase-2 (COX-2), interleukin (IL)-1β, IL-18, and P2X7 receptor (P2X7R), to name a few, and is found particularly in pancreatitis and pancreatic tumors [7–11]. In recent years, the role of ATP and its cognate receptors in the inflammatory process, has been recognized. To date, P2X7R is the most potent of the membrane receptors responsible for inflammasome activation and release of inflammatory cytokines. P2X7R is expressed primarily (though not exclusively) on cells of hematopoietic origin and appears to play an important role in macrophage/granulocyte function [12]. Upon transient stimulation, P2X7R behaves like a cation-selective channel permeable to Na^+^, K^+^, and Ca^2+^. However, sustained stimulation of P2X7R drives induction of NLRP3 inflammasome activation and IL-1β release. IL-1β activates the inflammatory cascade, including induction of COX-2, iNOS, and TNF-α [12, 13]. P2X7R activation also stimulates the release of other important lipid mediators, such as thromboxane (TX) B_2_ and leukotriene (LT) B_4_. P2X7R antagonists potently inhibit eicosanoid production as well as IL-1β release [12, 13]. Our laboratory and others have reported increased expression of P2X7R in pancreatic carcinoma [7–11]. Hence, P2X7R antagonism may lead to a wider range of anti-inflammatory effects than can COX-2 inhibitors [13]. A proinflammatory role for P2X7R was further supported by the demonstration that P2X7R knockout mice showed reduced inflammation in an arthritis model [13]. Different P2X7R antagonists have been used in animal models of inflammation and pain, and some of them are under extensive clinical trials. These molecules have successfully passed phase I clinical trials, and some of them are currently in phase II/III trials for rheumatoid arthritis, osteoarthritis, Crohn's disease, and chronic obstructive pulmonary disease [14].

In the present study, we i) evaluated the expression of P2X7R and other inflammatory markers in a genetically engineered mouse (GEM) model by next generation sequencing, ii) synthesized potent antagonists of P2X7R (i.e A438079 and AZ10606120), iii) determined optimal doses of A438079 and AZ10606120, iv) evaluated long term chemopreventive efficacy of both P2X7R inhibitors using PC GEM model, and v) studied the expression of P2X7R and various inflammasome related markers in untreated and treated tumors.

## RESULTS

### Expression analysis of P2X7R in LSL-Kras^G12D/+^ mice

**Figure 1:**
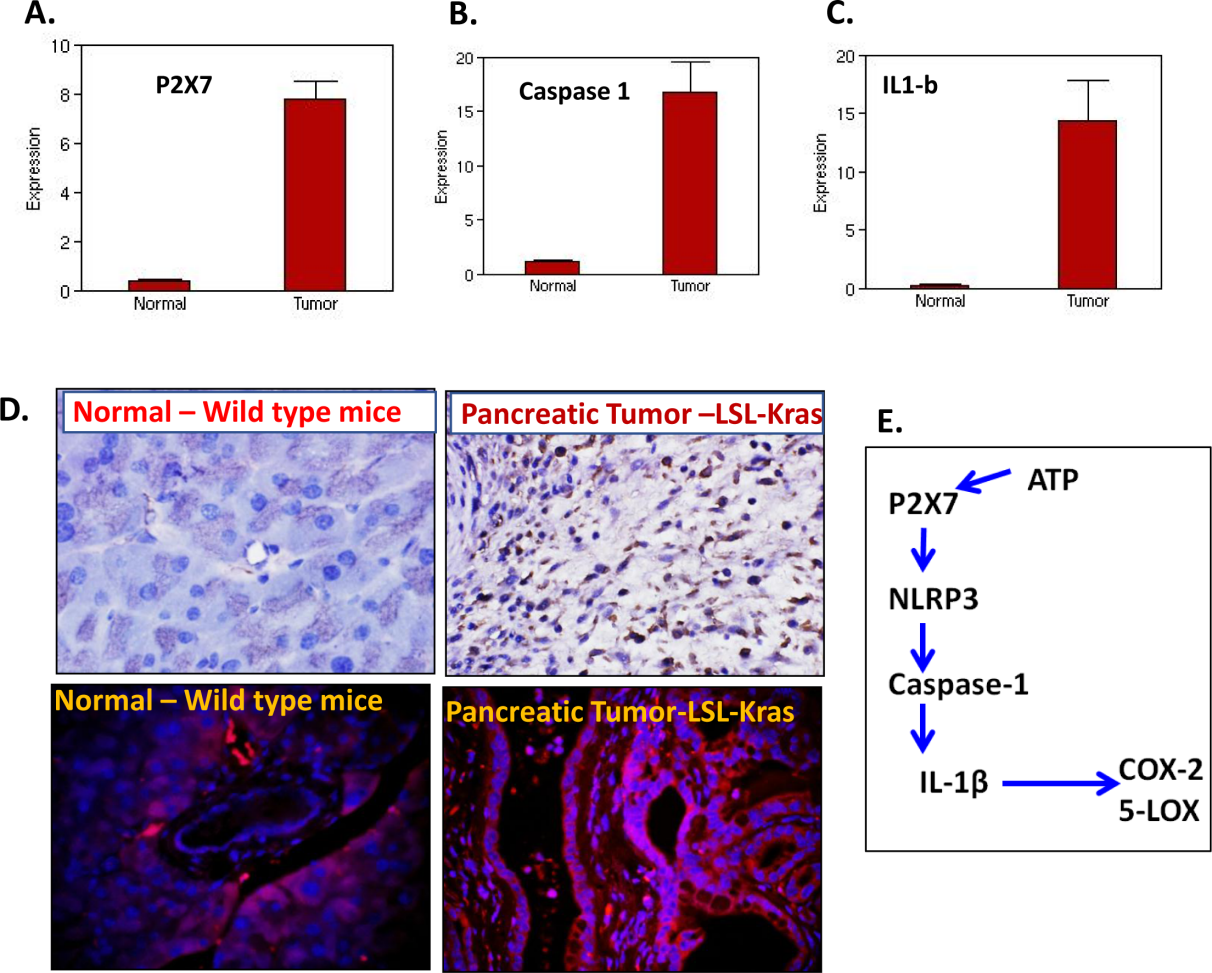
Expression of P2X7R and inflammasome markers in pancreatic tumors (**A**–**C**) NGS analysis showing mRNA overexpression of P2X7R (A), caspase-1 (B) and IL-1β in the pancreatic tumors from genetically engineered mice compared to normal pancreas from wild type mice. (**D**) IHC analysis of P2X7R expression in normal pancreas (upper left panel) and pancreatic tumor (upper right panel), IHF analysis of P2X7R expression in normal pancreas (upper left panel) and pancreatic tumor (upper right panel). (**E**) Schematic representation of P2X7R-NLRP-caspase-IL1β inflammasome cascade. Significant overexpression of P2X7R, caspase-1, IL-1β mRNA and P2X7R protein expressions were seen in the pancreatic tumors compared to normal pancreas.

Several lines of evidence from *in vitro* and *in vivo* studies support the pro tumorigenic role of P2X7 purinoceptor gene in various cancers, including human pancreatic cancer [11, 15]. To further understand the role of P2X7R and the inflammasome in pancreatic tumor progression, we carried out transcriptomic analysis of LSL-Kras pancreatic tumors by next generation sequencing. Our results show that P2X7R (~20-fold) (Figure 1A), its key inflammasome components: caspase-1 (15-fold) (Figure 1B), IL-1β (~45-fold) (Figure 1C), and in addition to (data not shown) IL-18 (~35-fold), IL-33 (~93 folds), TNF-α (~13-fold) and COX-2 (~41-fold) are increased in pancreatic tumors compared to normal pancreas. Further analyses of mouse PC tissues by immunohistochemistry and/or immunofluorescence (Figure 1D) suggest that P2X7R is a critical contributor to the progression of pancreatic tumor growth through inflammatory signaling (Figure 1E).

### Synthesis of A438079 and AZ10606120

**Figure 2:**
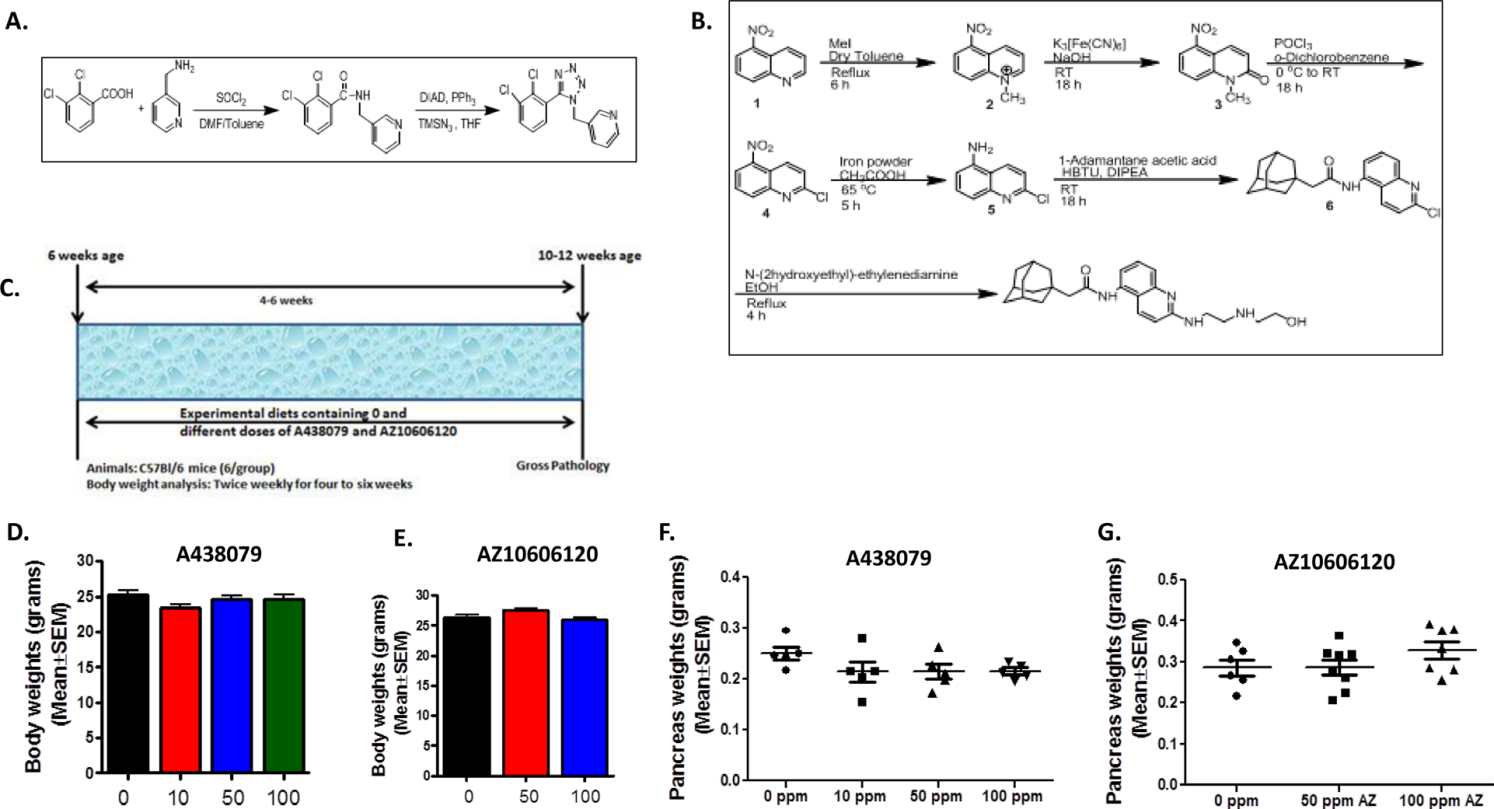
Chemical synthesis and dose optimization of P2X7R inhibitors A438079 and AZ10606120 (**A**) Synthesis of A438079. (**B**) Synthesis of AZ10606120. (**C**) Experimental design for MTD/dose optimization study in mice. (**D**) Body weights of mice treated with A438079 for six weeks. (**E**) Body weights of mice treated with AZ10606120 for six weeks. (**F**) Pancreas weights of mice treated with A438079 for six weeks. (**G**) Pancreas weights of mice treated with AZ10606120 for six weeks. X- axis: body weight or pancreas weight in grams; y-axis: doses of A438079 or AZ10606120 in ppm. No significant difference in body weights and pancreas weights were observed in the P2X7R inhibitors (A438079 and AZ10606120) treatment groups compared to untreated.

We synthesized P2X7R inhibitors A438079 and AZ10606120 for the MTD and chemoprevention efficacy studies from the procedures described in previous publications and the patent application filed by Jones, *et al*. (PCT International Publication Number WO 2009/106564 A2) (Figure 2A–2B). HPLC analysis was performed to verify the purity (>98%) of the agents (Supplementary Figure 1).

### MTD and the optimal dose of A438079 and AZ10606120

MTD and optimal non-toxic dose studies on both P2X7R antagonists were performed using C57Bl/6 wild type mice (Figure 2C). Based on the body weight gain and gross organ observations, up to 100 ppm A438079 or AZ10606120 did not have any effect on the body weights or pancreas weights (Figure 2D–2G) and there were no external signs of toxicity or any changes in other organ weights (data not shown). Hence, based on MTD studies, 50 and 100 ppm doses were selected for chemoprevention efficacy studies using both P2X7R inhibitors in the p48^Cre/+^-LSL-Kras^G12D/+^ GEM.

### Chemopreventive efficacy of AZ10606120 and A438079 in p48^Cre/+^-LSL-Kras^G12D/+^ mice

### General health of animals treated with AZ10606120 and A438079

**Figure 3:**
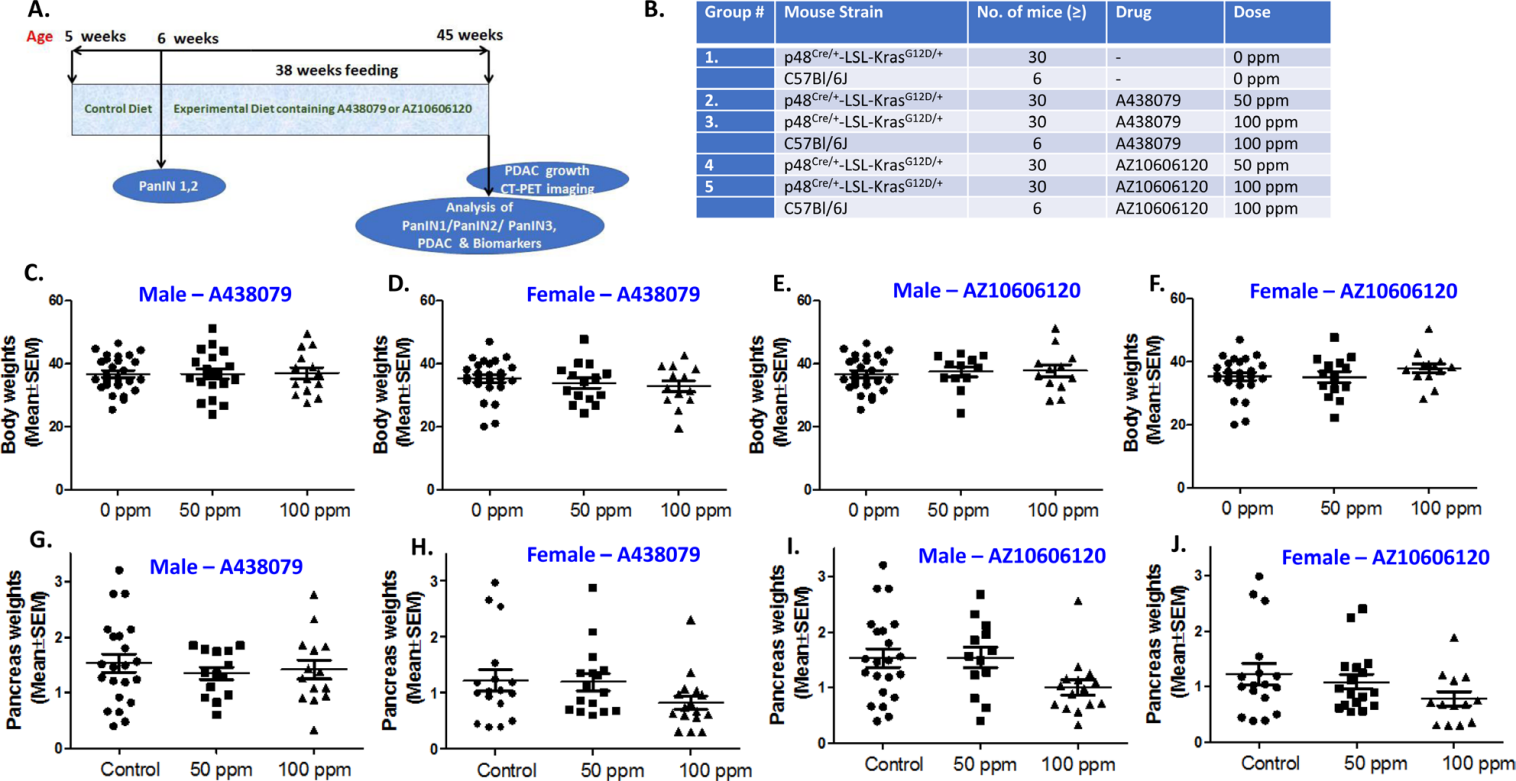
Chemoprevention of pancreatic cancer by P2X7R inhibitors (**A**) Experimental design to evaluate the chemopreventive and dose-response effects of AZ10606120 and A438079 in p48^Cre/+^-LSL-Kras^G12D/+^ GEM. (**B**) Doses for the efficacy of A438079 and AZ10606120 using GEM. (**C**) Effect of A438079 on body weight gain of male GEM at 45 weeks (41 weeks for 100 ppm). (**D**) Effect of A438079 on body weight gain of female GEM at 45 weeks (41 weeks for 100 ppm). (**E**) Effect of AZ10606120 on body weight gain of male GEM at 45 weeks (41 weeks for 100 ppm). (**F**) Effect of AZ10606120 on body weight gain of female GEM at 45 weeks (41 weeks for 100 ppm). (**G**) Pancreas weights of male GEM treated with A438079 at 45 weeks’ age (41 weeks for 100 ppm). (**H**) Pancreas weights of female GEM treated with A438079 at 45 weeks’ age (41 weeks for 100 ppm). (**I**) Pancreas weights of male GEM treated with AZ10606120 at 45 weeks’ age (41 weeks for 100 ppm). (**J**) Pancreas weights of female GEM treated with AZ10606120 at 45 weeks’ age (41 weeks for 100 ppm).

The experimental protocol for evaluating A438079 and AZ10606120 in pancreatic cancer progression is summarized in Figure 3A. p48^Cre/+^- and LSL-Kras^G12D/+^ mice were bred to generate p48^Cre/+^-LSL-Kras^G12D/+^ at our rodent barrier facility. Figure 3B summarizes the doses of A438079 and AZ10606120 that were tested in pancreatic ductal adenocarcinoma efficacy studies by dietary administration. Based on the MTD/optimal dose studies and clinical dose-relevance, test agents A438079 and AZ10606120 were used at 50 and 100 ppm in the AIN-76A diet to assess the pancreatic tumor inhibitory efficacy. After generating the required number of p48^Cre/+^-LSL-Kras^G12D/+^ mice, all the experimental groups for 0, 50 and 100 ppm A438079 and AZ10606120 were evaluated (Figure 3A, 3B). p48^Cre/+^-LSL-Kras^G12D/+^ mice fed AIN-76A or A438079 and AZ10606120 diets had steady body weight gain (Figure 3C–3F). As shown in Figure 3C–3F, there was no significant difference in body weight in the mice fed either AIN-76A or AIN-76A diets supplemented with 50 ppm A438079 and AZ10606120. At 45 weeks of age, control and low dose treatment mice were euthanized, whereas higher dose treated mice were euthanized at 41 weeks’ age (i.e. 4 weeks before actual termination schedule as the mice were not able to survive beyond this point, and the pancreatic tumor weights were recorded.

### Effect of A438079 and AZ10606120 diet on pancreatic tumor weight and PDAC Incidence in GEM

Pancreatic weight is a simple marker to assess the progression of a tumor. The pancreas weights of wild type mice at 45 weeks’ age were ~200-300 mg. An increase in pancreatic weight (0.8 g to 1.5 g, a range across the groups) was observed in p48^Cre/+^-LSL-Kras^G12D/+^ mice. As summarized in Tables 1 and 2 and Figure 3G–3J, A438079 and AZ10606120 treatment at higher doses caused a marginal decrease in the weight of pancreatic tumors in p48^Cre/+^-LSL-Kras^G12D/+^ mice. In GEM fed 50 or 100 ppm A438079, the mean pancreatic weights were 1.34 g and 1.41 g, respectively, in males, and 1.35 g and 0.73 g in females. In GEM fed 50 or 100 ppm AZ10606120, the mean pancreatic weights were 1.53 g and 1.09 g, respectively, in males and 1.12 g and 0.78 g, respectively, in females. Extensive histopathologic analysis of the pancreas using H&E-stained slides revealed no microscopic pathologic alterations in wild-type mice fed either AIN-76A or drug-supplemented diets. In contrast, dietary A438079 and AZ10606120 showed up to 60% incidence of PDAC (Tables 1 and 2). A438079 and AZ10606120 treatment at 50 ppm showed an increase in PDAC incidence in male mice whereas, in female mice, AZ10606120 treatment at 50 ppm showed reduced PDAC incidence (Tables 1 and 2). The higher doses of both agents were not considered for efficacy evaluation due to early euthanasia. About 45% of mice in higher doses of both agents were sick and had to be euthanized before 41 weeks’ age. Hence, the remainder of the mice were euthanized at 41 weeks’ age, i.e. 4 weeks before actual termination. Although histology was carried out for these mice and the data are presented in the summary tables, it cannot be compared with control or lower dose treatments.

**Table 1:** Effect of P2X7R inhibitors on the body weight, PanINs multiplicity and PDAC in LSL-Kras^G12D/+^ male mice

	Agent	No. of mice	BW Grams Mean	PanIN1 Mean ± SE	PanIN2 Mean ± SE	PanIN3 Mean ± SE	Total PanINs Mean ± SE	% Normal Mean ± SE	% Ca spread Mean ± SE	Ca incidence	Pan WT Grams Mean
1	Control	26	38.5	208 ± 25	106 ± 11	25 ± 6	339 ± 14	8.1 ± 2.9	24.3 ± 6.1	50%	1.56
2	A438079 50 ppm	19	36.7	105 ± 27	58 ± 13	27 ± 12	190 ± 17	8.2 ± 3.7	52.7 ± 10.2	60%	1.34
3^*^	A438079 100 ppm	17	38.7	173 ± 52	99 ± 29	34 ± 15	306 ± 32	3.2 ± 1.1	49.7 ± 13.9	61%	1.41
4	AZ10606120 50 ppm	17	37.4	168 ± 63	76 ± 22	27 ± 13	271 ± 33	12.5 ± 7.4	43.3 ± 15.1	63%	1.53
5^*^	AZ10606120 100 ppm	13	37.7	292 ± 64	97 ± 17	39 ± 26	428 ± 36	10.2 ± 6	12 ± 10.4	33%	1.09

^*^Mice in these groups were euthanized four weeks before actual termination schedule.

**Table 2:** Effect of P2X7R inhibitors on the body weights, PanINs multiplicity and PDAC in LSL-Kras^G12D/+^ female mice

	Agent	No. of Mice	BW Mean	PanIN1 Mean ± SE	PanIN2 Mean ± SE	PanIN3 Mean ± SE	Total PanINs Mean ± SE	% Normal Mean ± SE	% Ca Mean ± SE	Ca incidence	Pan WT Mean
1	Control	17	36.4	210 ± 39	78 ± 14	22 ± 7	310 ± 20	7.3 ± 2.6	25.6 ± 3.4	40%	1.04
2	A438079 50 ppm	17	36.3	209 ± 41	84 ± 13	37 ± 14	330 ± 23	2.0 ± 0.5	20.3 ± 3.8	50%	1.35
3^*^	A438079 100 ppm	17	36.1	378 ± 43	166 ± 20	13 ± 9	557 ± 24	9.0 ± 2.2	8.8 ± 4.1	25%	0.73
4	AZ10606120 50 ppm	17	35.1	334 ± 53	118 ± 16	33 ± 17	485 ± 29	6.5 ± 2.3	9.6 ± 3.0	27%	1.12
5^*^	AZ10606120 100 ppm	19	38.6	250 ± 74	86 ± 19	30 ± 18	366 ± 37	20.2 ± 3.4	11.5 ± 3.4	22%	0.78

^*^Mice in these groups were euthanized four weeks before actual termination schedule.

### Effect of A438079 and AZ10606120 on PanIN lesion progression and % carcinoma

Histological analysis showed 100% penetrance of pancreatic precursor PanIN lesions in the GEM fed AIN76A or drug-supplemented diets. The number of PanIN 1, 2, and 3 lesions in male GEM fed AIN76-A diet were (means ± SEM): 208 ± 25, 106 ± 11, and 25 ± 6, respectively; in the mice fed 50 ppm A438079, PanIN 1, 2, and 3 numbers were 105 ± 27, 58 ± 13, and 27 ± 12; and in mice fed 50 ppm AZ10606120 they were 168 ± 63, 76 ± 22, and 27 ± 13, respectively (Table 1). The number of PanIN 1, 2, and 3 lesions in female GEM fed AIN76A diet were 210 ± 39, 78 ± 14, and 22 ± 7, respectively; in the mice fed 50 ppm A438079, the numbers were 209 ± 41, 84 ± 13, and 37 ± 14; and in mice fed 50 ppm AZ10606120, they were 334 ± 53, 118 ± 16, and 33 ± 17, respectively (Table 2). The number of PanIN 3 lesions or carcinoma *in situ* was marginally increased in both drug-treated groups (Tables 1 and 2). Pancreas of male GEM fed AIN76 A diet showed a 24.3 ± 3.4 % (Table 1) incidence of PDAC within the pancreas, while in female mice it was a 25.6 ± 3.4 % (Table 2). The carcinoma percentage within the pancreas was significantly increased (up to 2-fold in males; Table 1) by both drugs in GEM. Female GEM treated with higher dose of A438079 and lower dose of AZ10606120 showed reduced carcinoma (Table 2). Although higher dose of AZ10606120 showed reduced carcinoma, due to early termination this group is not used for comparison (~45% of mice).

### Modulation of predictive specific signature marker(s) by A438079 and AZ10606120 in pancreatic cancer

**Figure 4:**
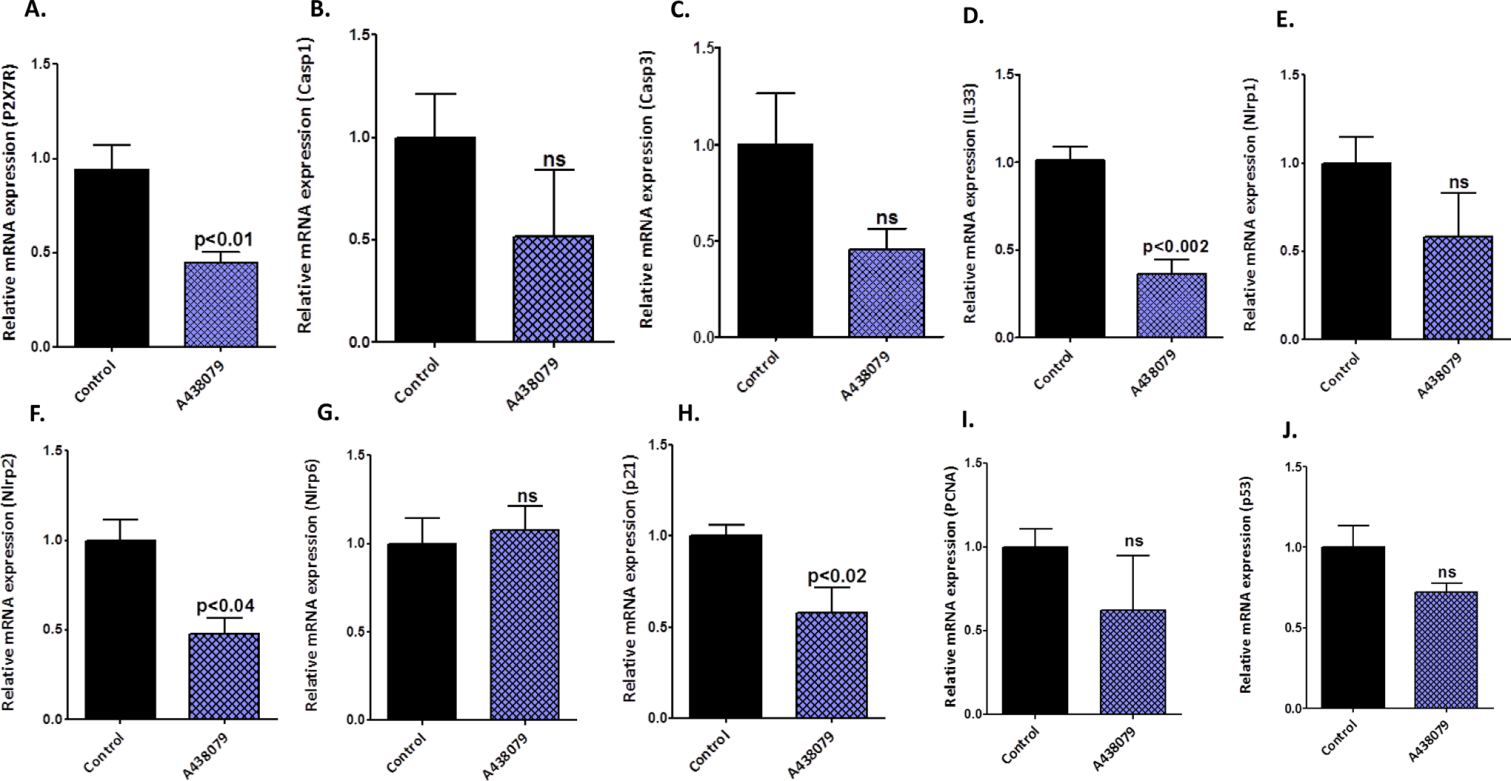
Biomarker modulation by A438079 in pancreatic tumors (**A**–**J**) Effect of A438079 (50 ppm) on mRNA expression of P2X7R (A), Caspase-1 (B), Caspase-3 (C), IL-33 (D), NLRP1 (E), NLRP2 (F), NLRP6 (G), p21 (H), PCNA (I) and p53 (J) in pancreatic tumors from male p48^Cre/+^-Kras^G12D/+^ mice (statistical analysis is performed by *t*-test using graph pad prism, Mean ± SE; *p* < 0.05; ns; non-significant).

**Figure 5:**
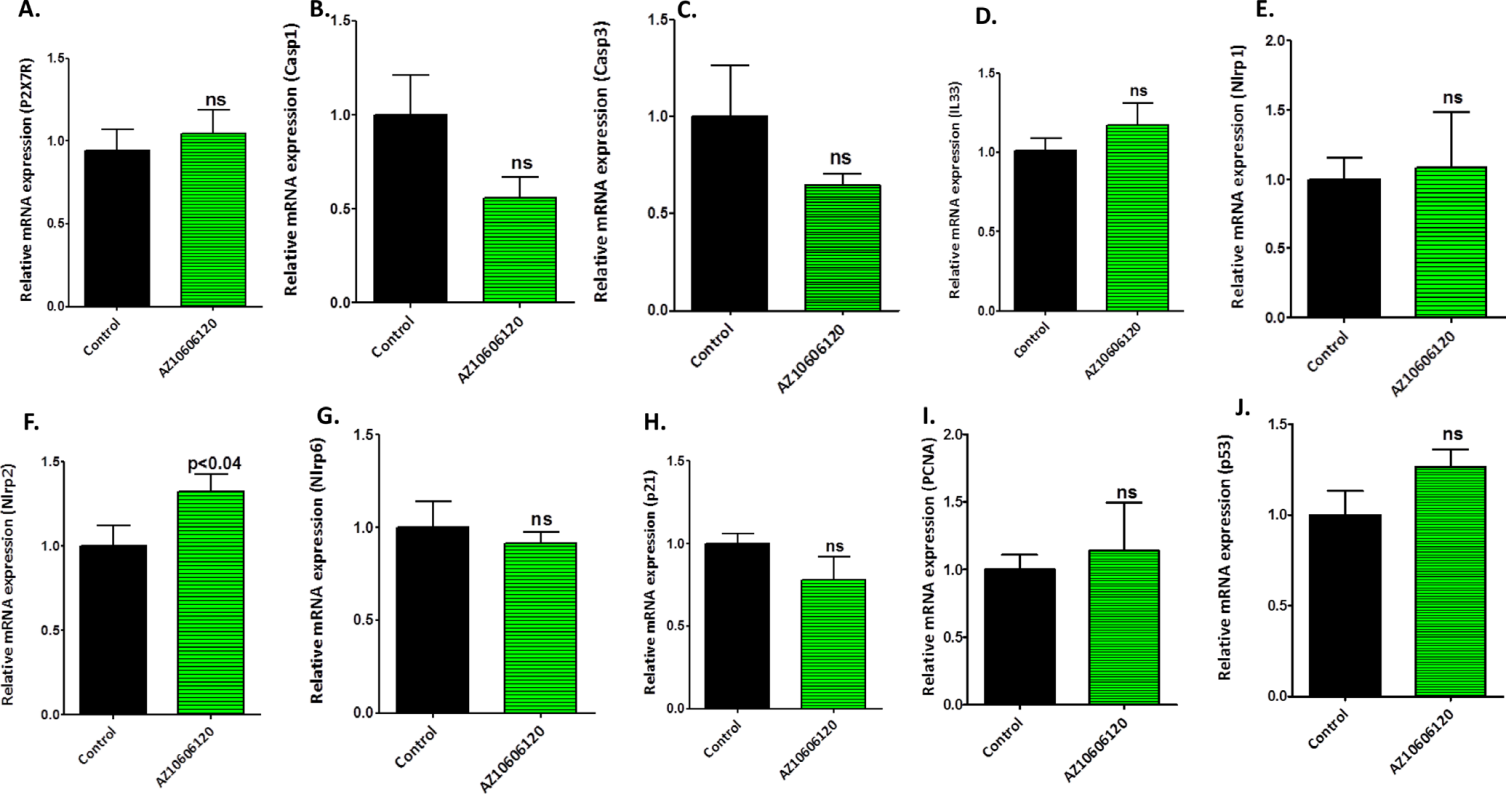
Biomarker modulation by AZ10606120 in pancreatic tumors (**A**–**J**) Effect of AZ10606120 (50 ppm) on mRNA expression of P2X7R (A), Caspase-1 (B), Caspase-3 (C), IL-33 (D), NLRP1 (E), NLRP2 (F), NLRP6 (G), p21 (H), PCNA (I) and p53 (J) in pancreatic tumors from male p48^Cre/+^-Kras^G12D/+^ mice (statistical analysis is performed by *t*-test using graph pad prism, Mean ± SE; *p* < 0.05; ns; non-significant).

**Figure 6:**
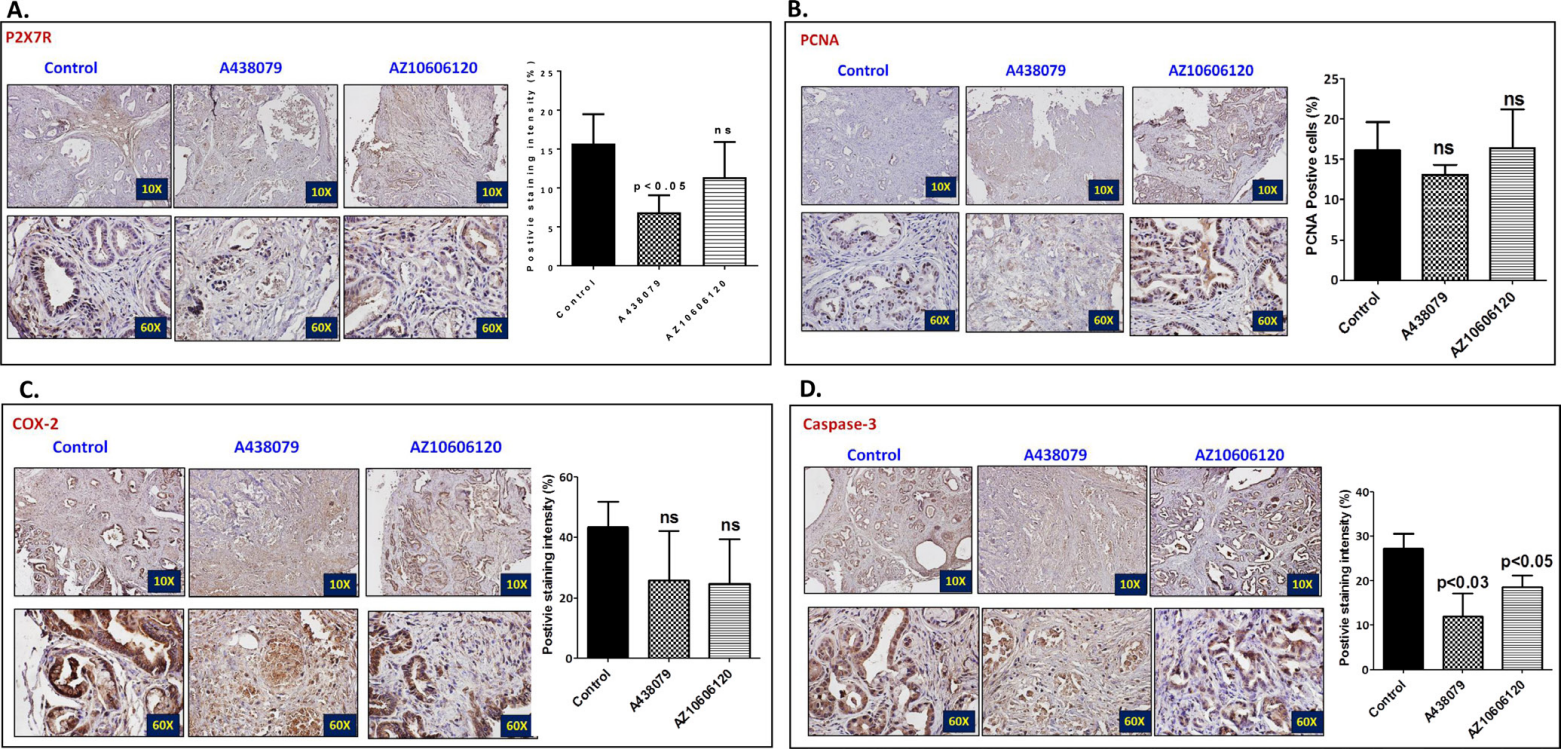
Effect of A438079 and AZ10606120 on expression of P2X7R, PCNA, COX-2 and caspase-3 (**A**–**D**) Effect of A438079 (50 ppm) and AZ10606120 (50 ppm) on P2X7R (A), PCNA (B), COX-2 (C) and caspase-3 (D) expressions in pancreatic tumors from male p48^Cre/+^-Kras^G12D/+^ mice at 45 weeks’ age as determined by immunohistochemistry. Histogram showing PCNA positive cells and P2X7, COX-2 and caspase-3 staining intensity (%).

**Figure 7:**
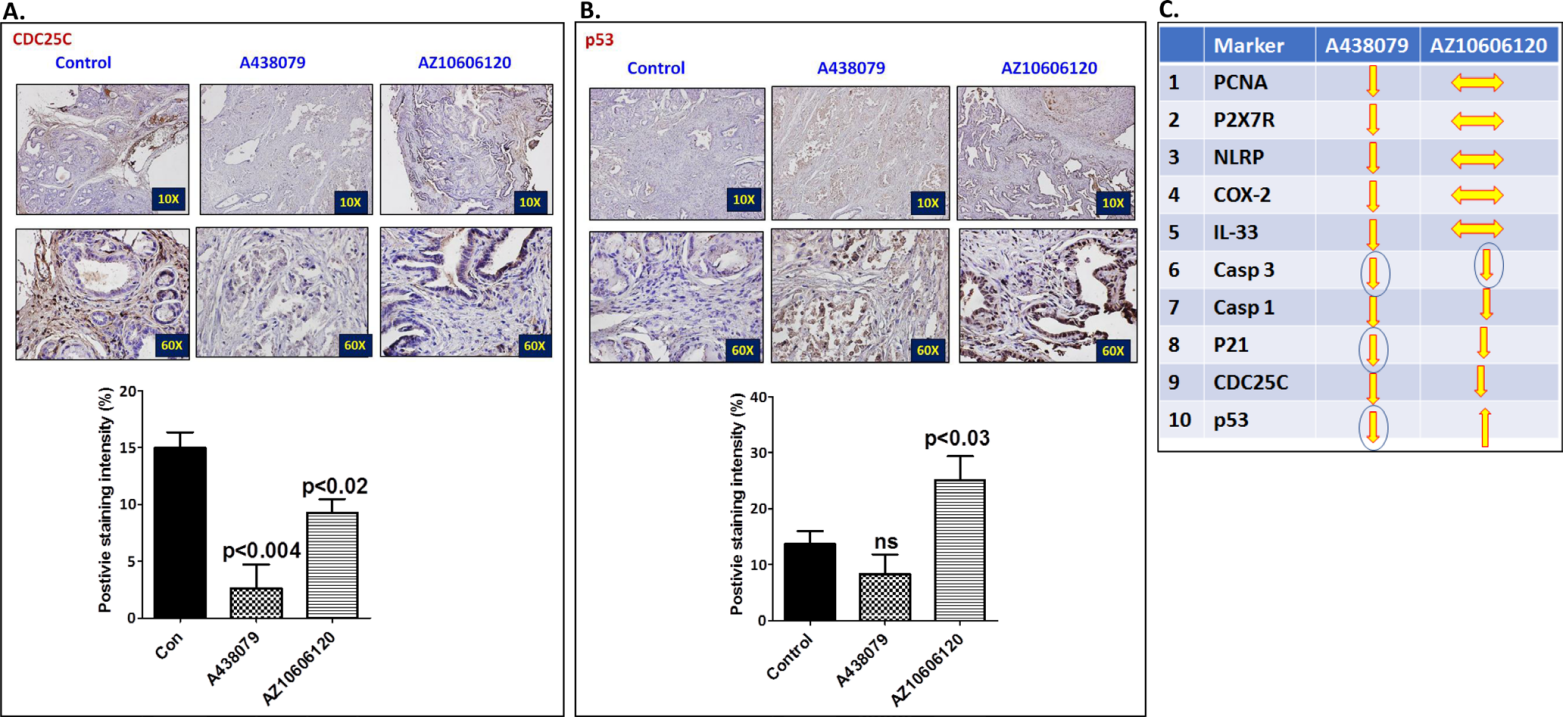
Effect of A438079 and AZ10606120 on expression of CDC25C and p53 (**A**–**B**) Effect of A438079 (50 ppm) and AZ10606120 (50 ppm) on CDC25C (A) and p53 (B) expressions in pancreatic tumors from male p48^Cre/+^-Kras^G12D/+^ mice at 45 weeks’ age as determined by immunohistochemistry. Histogram showing CDC25C and p53 staining intensity (%). (**C**) Summary of effect of P2X7R inhibitors on the biomarker expression (mRNA and protein) modulation in pancreatic tumors from male GEM as determined by real-time PCR and immunohistochemistry.

The pancreatic tumor tissues obtained from efficacy studies were used to determine the predictive signature markers and dose response effects of A438079 and AZ10606120. Signature markers associated with tumor growth using the pancreas from wild type mice and 45-week-old p48^Cre/+^-LSL-Kras^G12D/+^ mice were analyzed by transcriptome analysis (Figure 1). Furthermore, we completed relevant biomarker analyses of the pancreatic tumor tissues from lower dose untreated and treated male mice to compare the effects of P2X7R inhibitors on tumor growth and their responses on signature markers in comparison to untreated mouse tumors by real-time PCR analysis and immunohistochemistry (Figures 4, 5, 6, 7). Dietary A438079 significantly reduced mRNA expressions of P2X7R, IL-33, NLRP3 and p21 while non-significant reduction was seen for caspase-1, caspase-3, NLRP-1, PCNA and p53 in the pancreatic tumor tissues (Figure 4). Dietary AZ10606120 significantly increased mRNA expressions of NLRP-2 (Figure 5). A non-significant decrease was seen for caspase-1, caspase-3, and p21 with increase in p53 in the pancreatic tumor tissues (Figure 5). A438079 had no effects on mRNA expression of NLRP-6 whereas AZ10606120 did not show significant change in the mRNA expressions of IL-33, NLRP-1, NLRP-6 and p21 (Figures 4, 5). Immunohistochemistry results revealed that A438079 significantly reduced protein expression of P2X7R, CDc25c and caspase-3 while a non-significant decrease was seen for p53, PCNA and COX-2 (Figures 6, 7). Immunohistochemistry results revealed that AZ10606120 significantly reduced the protein expression of CDc25c and caspase-3 while a non-significant decrease was seen for P2X7R and COX-2 (Figures 6, 7). AZ10606120 had no effects on PCNA but significantly increased p53 (Figures 6, 7).

## DISCUSSION

The etiology of PDAC and laboratory investigations have demonstrated that inflammation represents an important role in its carcinogenesis. P2X7R is responsible for inflammasome activation and release of inflammatory cytokines like IL-1β release through NLRP3 and caspase-1 activation (Figure 1E). IL-1β activates the inflammatory cascade (Figure 1E), including induction of COX-2, iNOS, and TNF-α [12, 13]. Several lines of evidence from *in vitro* and *in vivo* studies support the pro-tumorigenic role of the P2X7 purinoceptor gene in various cancers, including human pancreatic cancer [11, 15]. To further understand the role of P2X7R and the inflammasome in pancreatic tumor progression, we carried out transcriptomic analysis of the p48^Cre-^LSL-Kras^G12D/+^ pancreatic tumors by Next Generation Sequencing. Our results show that the P2X7R and its key inflammasome components: IL-1β, IL-18, IL-33, and caspase-1, as well as several IL-1β-dependent genes such as COX-2 and tumor necrosis factor TNF-α, are highly overexpressed (Figure 1). Analyses of mouse PC tissues suggest that P2X7R is a critical contributor to the progression of pancreatic tumor growth (Figure 1). In a recently published study, we showed that atorvastatin-induced delay of PDAC progression was associated with down regulation of the P2X7 receptor in p48^Cre-^LSL-Kras^G12D/+^ mice [11]. These studies clearly underscore the importance of P2X7 receptor/inflammasome in PDAC progression and suggested that P27XR would be a potential target in pancreatic cancer prevention.

Different P2X7R antagonists have been used extensively in animal models of inflammation and pain, and some of them are under extensive clinical trials. Several P2X7 antagonists have been shown to be well tolerated in Phase I and II studies [19]. More than 30 clinical studies have been performed so far to test the efficacy of P2X7R blockade in osteoarthritis, rheumatoid arthritis, chronic obstructive pulmonary disease, and Crohn's disease [20–27]. AZ10606120, *N*-[2-[[2-[(2-hydroxyethyl)amino]ethyl]amino]-5-quinolinyl]-2-tricyclo[3.3.1.13,7]decylacetamide dihydrochloride (AZ10606120) and 3-[[5-(2,3-Dichlorophenyl)-1H-tetrazol-1-yl]methyl] pyridine hydrochloride (A438079) are P2X7 receptor antagonists, selected for further development because of their improved selectivity for P2X7R, their potential to block P2X7R-dependent inflammasome activities [14, 15, 19], and their demonstrated anti-tumor activities (>50%) in preclinical models [28]. The overall objective of the present study was to develop specific inhibitors of (P2X7R) for pancreatic cancer prevention in preclinical models.

We first established the optimal dose/nontoxic dose in mice and then assessed the pancreatic cancer prevention efficacy in the p48^Cre/+^-LSL-Kras^G12D/+^ genetically engineered mouse model of pancreatic cancer. Both agents did not show overt toxicities when tested up to 100 ppm in the dose optimization studies. In the efficacy studies, lower doses of both agents (A438079 and AZ10606120) in male mice increased carcinoma incidence. Carcinoma spread was increased by about 2-fold (Tables 1 and 2). Although lower doses of AZ10606120 in female mice showed a 32% inhibition of carcinoma incidence, there was an increase in pancreatic tumor weights (Table 2). However, higher doses of both drugs showed increase in mortality due to tumor progression effects which lead to early termination of the mice by 4 weeks. In addition, we evaluated drug-induced modifications in inflammasome signal pathways and critical biomarkers of efficacy. There was no clear indication on the effects of both agents on the inflammasome markers and their correlation with efficacy. A clear effect on molecular alteration was not seen with both the drugs (Figures 4, 5, 6, 7).

It is evident from our studies that P2X7R is overexpressed in the pancreatic tumors compared to normal pancreas and is correlated with increased expression of inflammasome markers. However, dietary administration of P2X7R antagonists failed to show chemopreventive effects. Although exact reasons are unknown, some of the recent studies support these results. In P2X7R-deficient mice, tumor growth and metastatic spreading were accelerated strongly, compared with wild-type mice [20]. Intra-tumoral IL-1β and VEGF release were drastically reduced, and inflammatory cell infiltration was abrogated nearly completely [28]. Another study, in the therapeutic context, evaluated the effects of AZ10606120 using mouse orthotropic xenograft studies [29]. PancTu-1 Luc cells were orthotopically transplanted into nude mice and tumor growth was followed noninvasively by bioluminescence imaging [29]. Although AZ10606120-treated mice showed reduced bioluminescence compared to saline-treated mice there was no significant effects on invasion and metastasis development, although *in vitro* have shown clear effects of the inhibitor on PancTu-1 Luc cell migration [29]. From this study, Giannuzo *et al* summarize that despite the high P2X7R over-expression of PancTu-1 Luc cells, the invasion and metastatic behavior of the undifferentiated tumors formed by these cells *in vivo* is too aggressive to be blocked only by this inhibitor alone [29]. Since the present study involved GEM models that show stepwise progression of PanIN lesions to ductal adenocarcinoma and that both P2X7R inhibitors failed to show any tumor inhibitory effects in line with other reports [29], further studies are warranted to determine the exact reasons for the failures. Despite safety profiles, P2X7R inhibitors showed disappointing clinical efficacy effects in arthritis and COPD [20] whereas somewhat encouraging results were obtained for Crohn's disease [20, 24, 27]. Several *in vitro* and *in vivo* animal studies clearly show that P2X7R is involved in the inflammatory process through NLRP3-caspase1-IL1β cascade, targeting P2X7R by antagonists provided controversial results.

Hence, caution is needed when P2X7R inhibitors are being used as anti-inflammatory agents for the treatment of arthritis, COPD or Crohn's diseases, especially if the same patients are at risk for colon and pancreatic cancers.

## MATERIALS AND METHODS

### Mouse model

Generation of p48^Cre-^LSL-Kras^G12D/+^ mice expressing the activated KrasG12D oncogene has been described previously [16]. All animal research was performed under the animal protocols approved by the University of Oklahoma Health Sciences Center (OUHSC) Institutional Animal Care and Use Committee (IACUC). Animals were housed in ventilated cages under standardized conditions (21°C, 60% humidity, 12-h light/12-h dark cycle, 20 air changes/h) in the University rodent barrier facility. Mice were allowed *ad libitum* access to the respective diets and to automated tap water purified by reverse osmosis. The primary antibodies used were PCNA, P2X7R, COX-2, caspase-3, cdc25 and p53 from Santa Cruz Biotechnology (Santa Cruz, CA) /Cell Signaling.

### Breeding and genotyping analysis

LSL-Kras^G12D/+^ and p48^Cre/+^ mice were maintained in a C57BL/6 heterozygous genetic background. LSL-Kras^G12D/+^ and p48^cre/+^ mice were bred and the offspring of activated p48^Cre/+^-LSL-Kras^G12D/+^ and C5BL/6 wild type mice were generated at required quantities. Briefly, genomic DNA was isolated from tail tissue samples using the mini-prep kit (Invitrogen, Carlsbad, CA). Polymerase chain reaction (PCR) was performed for Kras and Cre genes using the following conditions: denaturation at 95°C for 5 min, followed by 35 cycles at 95°C for 1 minute, 600C for 1 minutes, and 72°C for 1 minute. Oligonucleotide primer sequences used were as follows: Kras 5′-CCTTTACAAGCGCACGCAGAG-3′ sense, 5′-AGCTAGCCACCATGGCTTGAGTAAGTCTGCA-3′ anti-sense; and p48Cre 5′-ACCGTCAGTACGTGAGATATCTT-3′ sense and 5′-ACCTGAAGATGTTCGCGATTATCT-3′ antisense. PCR products were separated on a 2% agarose gel. Successful recombination yields were 550 and 350-bp products for Kras and Cre genes respectively [16]. The genotype of each pup was confirmed by tail DNA extraction and PCR.

### Whole transcriptome analysis (WTA) methodology

Next Generation Sequencing (NGS)-based WTA expression analysis was performed using Next Generation Sequencer. Total RNA was extracted from the pancreatic tissues with Totally RNA kit (Ambion), followed by mRNA purification (Ambion kit). The quality of the purified mRNA was tested with a Bio-analyzer (OUHSC Microgen core facility). Samples with RNA integrity (RIN) 5 to 9 were sent for Next Generation Sequencing at the OUHSC laboratory for genomics and bioinformatics Microgen core facility (http://www.microgen.ouhsc.edu/). The readouts were analyzed with GeneSifter software; (Geospiza; http://www.geospiza.com/), available at the Microgen core.

***Data analysis***. The data analysis was done by mapping all reads generated sequentially against whole transcriptome (mRNA) Sanger data base/reference sequences built against mice. The number of reads per mRNA was determined and used as the expression level for that particular mRNA. Resulting reads/counts were normalized by total sequences for each sample, deriving the “cpm” or counts per million sequences.

***Validation of methods***. Further confirmation/validation studies involved Immuno-histochemistry to quantitate the protein levels of biomarkers that were identified for specific mRNAs observed through transcriptome analysis in normal pancreas vs PDAC.

### Experimental diets/preparation/quality control

Adequate and controlled nutrition for laboratory animals is essential to achieve reproducibility of data. Because of variations in NIH-07 or Purina Lab Chow, we used a semipurified diet based on modified AIN-76A (Casein, 20%; Corn Starch, 52%; Dextrose, 13%; Corn oil, 5.0%; Alphacel/cellulose, 5.0%; DL-Methionine, 0.3%; Mineral mix AIN, 3.5%; Vitamin mix, AIN, 1.0%; and Choline bitartrate, 0.2%) [17, 18]. All experimental semi-purified diets were formulated and prepared at the research diet preparation core. All ingredients of the semipurified diet were purchased from the same source (Bioserv, NJ) throughout the study. All diets were mixed thoroughly so that all micronutrients were uniformly distributed in the diet. In order to assure that test agents were uniformly distributed in the diet, the agents were pre-mixed with a small quantity of control diet in a food mixer, added to pre-weighed amount of control diet in a Hobart Mixer, and mixed thoroughly for about 45 minutes. Both control and experimental diets were prepared weekly and stored at 4°C in the cold room. Mice were allowed *ad libitum* access to the respective diets and to automated tap water purified by reverse osmosis.

### Synthesis of AZ10606120 and A438079

The following synthetic procedures were developed for the bulk synthesis of AZ10606120 and A438079 at the College of Pharmacy, OU Health Sciences Center, from the procedures described in previous publications and the patent application filed by Jones, *et al*. (PCT International Publication Number WO 2009/106564 A2) (Figure 1). Briefly, the 2,3 dichloro benzoic acid was reacted with excess of thionyl chloride to give corresponding acid chloride in quantitative yield. This acid chloride was reacted with equivalent amount of amine to give 2,3-dichloro-N-pyridin-3-ylmethylbenzamide. The 2,3-dichloro-N-pyridin-3-ylmethylbenzamide was then converted to 3-[5-(2,3-Dichloro)-tetrazol-1-ylmethyl]pyridine (A438079) using Mitsunobu conditions. We obtained 33.98 % yield of A438079 (Figure 1). Briefly, for AZ10606120 synthesis, 5-nitro quinoline was reacted with Methyl Iodide in dry toluene to give N-Methyl-5-nitroquinoline. N-Methyl-5-nitroquinoline was converted to 1-Methyl-5-nitro-carbostyril with Potassium ferricyanide and sodium hydroxide in water. This carbostyril was refluxed with excess of Phosphorous oxychloride (POCl3) to obtain 2-Chloro-5-nitro quinoline after silicagel purification. The reduction of nitroquinoline by using SnCl2/NaBH4 gave 2-Chloro-5-aminoquinoline. This aminoquinoline was reacted with 1-adamantane acety chloride to give N-(2-Chloroquinolin-5-yl)-2-(adamantyl)acetamide. The amination of N-(2-Chloroquinolin-5-yl)-2-(adamantyl)acetamide with N-(2-hydroxyethyl)-ethyelenediamine gave N-(2-(2-(2-Hydroxyethylamino)quinolin-5-yl)-2-(adamantyl)acetamide (AZ10606120) in 83% yield (Figure 1). Purity was determined by HPLC (Supplementary Figure 1).

### Determine the MTD of AZ10606120 and A438079 in mice

The MTD is defined as the highest dose administered in the diet that causes no significant (generally not more than 10%) weight decrement as compared to the appropriate control diet and does not produce mortality or any external signs of toxicity that would be predicted to shorten the natural life span of the animal. Briefly, at 7 weeks of age, male mice in each group received their respective control and experimental diets containing one of the two or three dose levels of AZ10606120 and A438079 until termination of the study, i.e., after 6 weeks on experimental diets (Figure 2C). Body weights and symptoms of toxicity were recorded weekly for 6 weeks. All organs were examined grossly for any abnormalities.

### Bioassay method for efficacy testing

The experimental design to test the efficacy of AZ10606120 and A438079 is shown in Figure 3A, 3B. Briefly, after breeding the required quantities of Kras activated LSL-Kras^G12D/+^ mice (male and female), at 5 weeks of age the mice were divided into the appropriate treatment groups consisting of 24–30 mice/group. In addition, 6 C57Bl/6 were included in the control and 100 ppm dose groups which served as a quality control for the expected background pancreatic cancer tumor incidence in this strain of mice. As shown in Figure 2A–2B, AZ10606120 and A438079 were administered by diet at the PanIN stage (6 weeks of age). All animals were weighed every week for the first 10 weeks, then every four weeks throughout the remainder of the experiment. Mice at 45 weeks of age were killed by CO_2_ euthanasia, and PanIN lesions and PDAC were evaluated histopathologically.

### Efficacy endpoints

PanIN lesion multiplicity (PanIN1, PanIN2, PanIN3), ductal adenocarcinoma (PDAC) incidence, pancreatic tumor weights, PDAC spread/pancreas, dose-response, and efficacy effects.

### Euthanasia, necropsy, and pancreatic tissue processing

CO_2_ euthanasia was the method of choice because of its rapid depressant and anesthetic effects. The animal was placed in a specially designed chamber, which is connected to a compressed CO_2_ cylinder. Inflow to the chamber can be regulated precisely with compressed CO_2_.

***Pancreatic tissue processing***: Briefly, all organs were examined grossly and pancreas, liver and lung were frozen for future analysis. Pancreas was immediately preserved in 10% buffered formalin. Before fixing the pancreas in buffered formalin, half of the pancreatic tissue (head to tail) was harvested and snap frozen in liquid nitrogen and then stored at −80°C for various biomarker analyses.

### Histopathological evaluation of pancreatic lesions

Multiple sections (step cuts) were made on each pancreatic tissue or organ in which a metastasis may be anticipated. The tester identified all blocks and microscopic slides by reference to the animal's specific identification number and preserved them. All the tissue sections were stained for H&E for histological analysis. The pathologist recorded, documented, and reported all microscopic findings, including all abnormalities, lesions, neoplasms, metastatic tumors, and their anatomic location. Diagnosis of pancreatic tumors was based on criteria outlined in our previously published articles and summarized below [11, 16].

### Efficacy evaluation - statistical analysis of tumor data

Mean body weight gain was compared between the dietary treatments. ANOVA was used to compare the effect of various levels of test compounds on body weights. PDAC incidence and PanINs multiplicity among the groups were compared to determine any significant differences due to the treatment regimens. At the termination of the experiment, the total number of PDAC-bearing animals with respect to the total number of animals at risk in each group (tumor incidence) was tested by the Armitage's Chi-Square method. Since some animals died during the course of the experiment; a Censored Chi-Square test provided compute probabilities and significance. PanINs multiplicity (total number of non-invasive lesions per pancreas) was also calculated for each group and the significance of the difference between the two dose levels and among various test agents was calculated. The data were analyzed using Graph Pad Prism 5.1 Software.

### Quantitative real-time PCR analysis

Total RNA from tumor samples was extracted using RNA Kit for isolation of total cellular RNA (Invitrogen) as per the manufacturer's instructions. Equal quantities of DNA-free RNA were used for reverse transcription reactions for making cDNA using SuperScript reverse transcriptase (Invitrogen). The real-time PCR was carried out in a 25-μL reaction volume using 3 μL of a 1:10 cDNA dilution containing SYBR Green master mix (BioRad) and individual primers for each of the following genes - caspase-3, caspase-1, IL-33, NLRP-1, NLRP-2, NLRP-6, P2X7R, p21, p52, and PCNA – were run as individual reactions (Supplementary Table 1). All PCRs were done in a Biorad iCycler iQ real-time PCR detection system. The fluorescence threshold values (Ct) were calculated. Relative mRNA levels were assessed by standardization to glyceraldehyde phosphate dehydrogenase (GAPDH). Results are expressed as a fold difference in gene expression.

### Immunohistochemistry

Five-μm fixed sections were incubated with primary antibodies in a hybridization chamber for 1 h at room temperature or overnight at 4°C. The primary antibodies used were PCNA, P2X7R, COX-2, caspase-3, cdc25, and p53. Following incubations with primary antibody, sections were incubated for 1 h with anti-mouse or anti-rabbit secondary antibodies, as appropriate for each primary antibody, then visualized with diaminobenzidine (DAB) and counterstained with Hematoxylin for IHC. Slides were observed under an Olympus microscope 1×701 and digital computer images were recorded with an Olympus DP70 camera.

## SUPPLEMENTARY MATERIALS FIGURE AND TABLE



## Abbreviations

PC: Pancreatic cancer
PanIN: pancreatic intraepithelial neoplasia
PT: pancreatic tumor
P2X7R: Purinergic receptor P2X ligand-gated ion channel 7
NLRP: NOD-like receptor pyrine domain containing
IL: Interleukin
TNF: tumor necrosis factor
COX-2: cyclooxygenase-2
PanIN: pancreatic intraepithelial neoplasia
PDAC: pancreatic ductal adenocarcinoma
IHC: immunohistochemistry
RT-PCR: real-time polymerase chain reaction
GEM: genetically engineered mice
MTD: maximum tolerable dose
PCNA: proliferating cell nuclear antigen
CCPDD: Center for Cancer Prevention and Drug Development
H&E: Hemotoxylin and Eiosin
LOX: lipoxygenase
TNF: tumor necrosis factor
NGS: next generation sequencing
