# Supplementary material for: Lack of chemopreventive effects of P2X7R inhibitors against pancreatic cancer
Source: Oncotarget. 2017 Oct 26;8(58):97822–34. doi: 10.18632/oncotarget.22085 (PMC5716694; doi:10.18632/oncotarget.22085)
Supplement: Supplementary file 1 [file oncotarget-08-97822-s001.pdf]

Lack of chemopreventive effects of P2X7R inhibitors against pancreatic cancer

SUPPLEMENTARY MATERIALS

A. B.

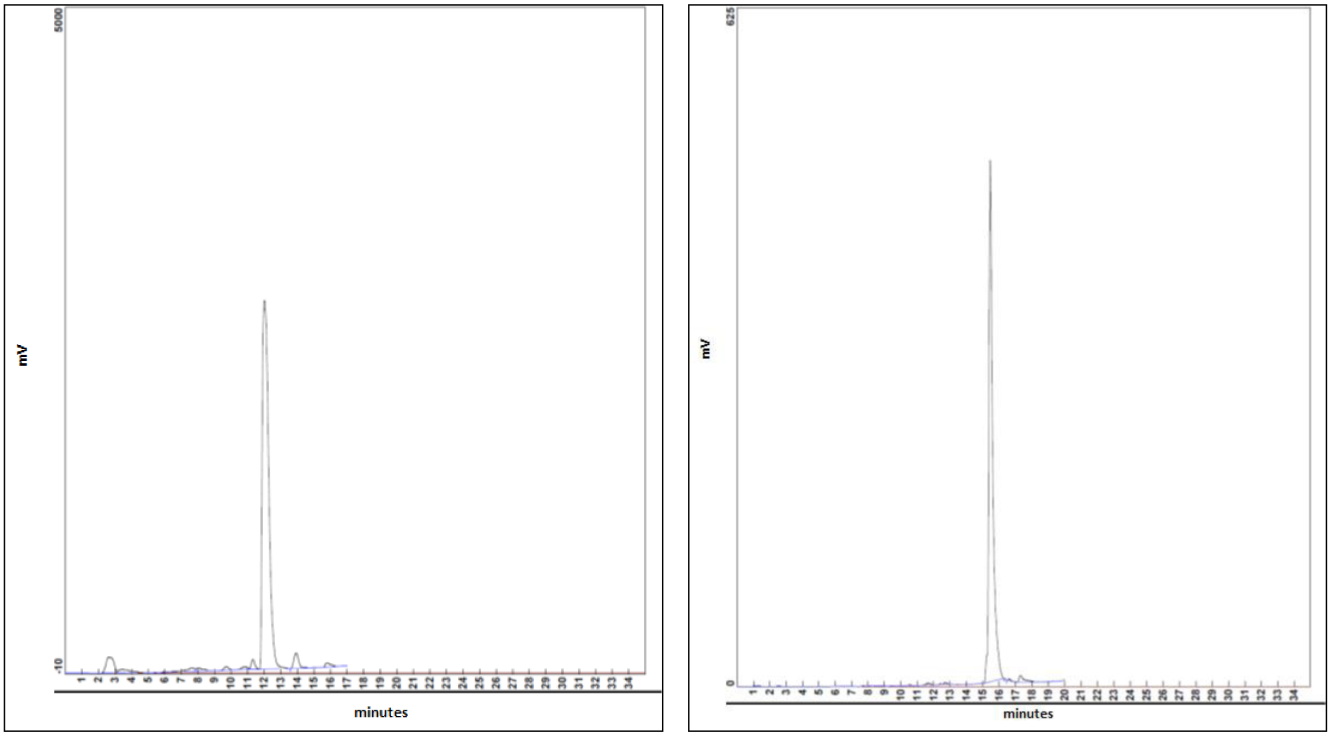

Supplementary Figure 1: (**A–B**) Purity analysis of A438079 (A) and AZ10606120 (B) by HPLC.

Supplementary Table 1: List of primers for determining mRNA expression in the pancreatic tumors

Il33 F	5′-TGAGACTCCGTTCTGGCCTC-3′
Il33 R	5′-CTCTTCATGCTTGGTACCCGAT-3′
Casp3 F	5′-GGCTTGCCAGAAGATACCGGT-3′
Casp3 R	5′-GCATAAATTCTAGCTTGTGCGCGTA-3′
Casp1 F	5′-AGATGCCCACTGCTGATAGG-3′
Casp1 R	5′-TTGGCACGATTCTCAGCATA-3′
PCNA F	5′-TAAAGAAGAGGAGGCGGTAA-3′
PCNA R	5′-TAAGTGTCCCATGTCAGCAA-3′
Tp53 F	5′-TGAAACGCCGACCTATCCTTA-3′
Tp53 R	5′-GGCACAAACACGAACCTCAAA-3′
NLRP1a F	5′-ATGTGGACCCAACCTTCAAA-3′
NLRP1a R	5′-GTACGTGCTCCTGGAAAGGT-3′
NLRP1b F	5′-CCCAGCACAAGACTCCACTT-3′
NLRP1b R	5′-CCAACCACCATGTGACTCTG-3′
NLRP2 F	5′-GACGTCCTGAGAAAGCTGGA-3′
NLRP2 R	5′-GGTCCATTGTCAGCTTGTTG-3′
NLRP6 F	5′-GCAGACGAGCTGCCTACTTT-3′
NLRP6 R	5′-GCTCCTGGTAACAGCTCCTG-3′
P2X7R F	5′-GCACGAATTATGGCACCGTC-3′
P2X7R R	5′-CCCCACCCTCTGTGACATTCT-3′
p21 F	5′-CGAGAACGGTGGAACTTTGAC-3′
p21 R	5′-TCCCAGACGAAGTTGCCCT-3′
